# Supplementary material for: Estimation of the Local Concentration of the Markedly Dense Hydroxyl Radical Generation Induced by X-rays in Water
Source: Molecules. 2022 Jan 18;27(3):592. doi: 10.3390/molecules27030592 (PMC8840408; doi:10.3390/molecules27030592)
Supplement: Supplementary file 1 [file molecules-27-00592-s001.zip › molecules-1483407-supplementary.pdf]

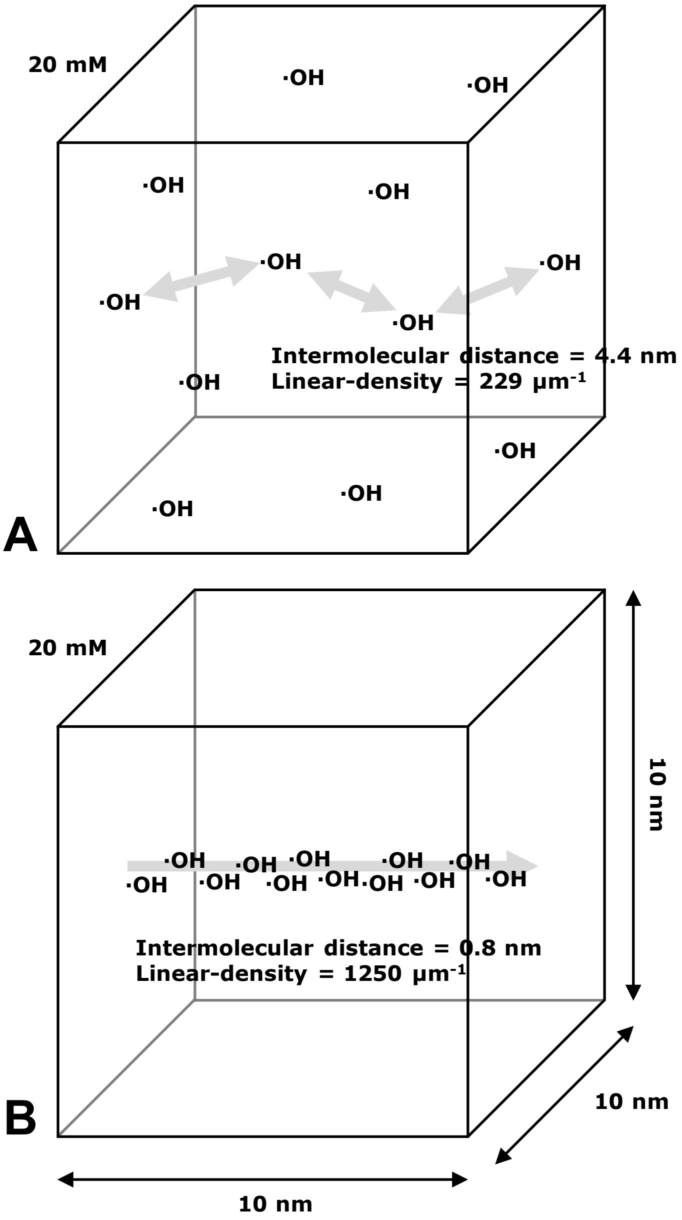

Figure S1. Conceptual images of uniform and localized distribution of molecules in a certain volume. (**A**) Twelve •OH were uniformly distributed in a 1000-nm^3 volume. (**B**) Twelve •OH were localized on a linear beam track. The concentration of •OH in both A and B is 20 mM. The linear-density, which is defined as the number of molecules aligned on the linear unit of distance, is the reciprocal of the inter-molecular distance of the subjected molecules at a certain concentration.

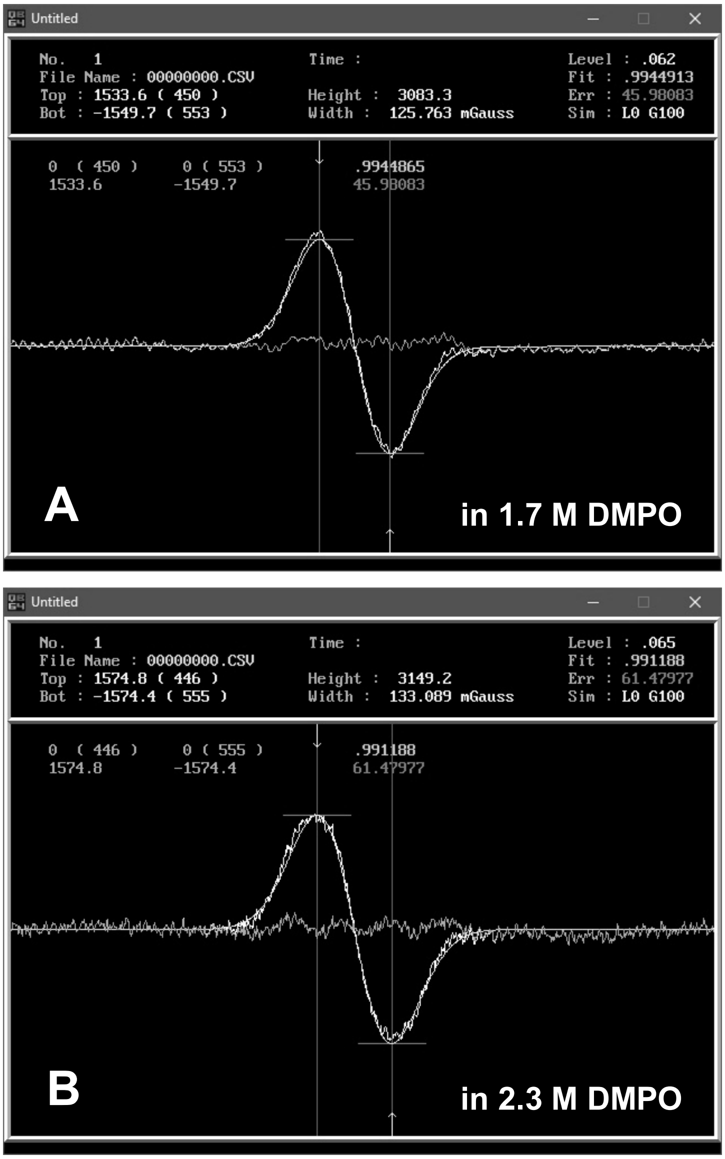

Figure S2. Display windows of the in-house line fitting software. A calculated Gaussian line was fitted on the 2nd line from the lower field of the 4-line EPR spectrum of DMPO-OH in (**A**) 1661 mM and (**B**) 2278 mM DMPO water solution by X-ray irradiation. The white line indicates the experimental spectrum.
